# DNA Engineering and Hepatitis B Virus Replication

**DOI:** 10.3389/fmicb.2021.783040

**Published:** 2021-11-11

**Authors:** Chun-yang Gan, Jing Cui, Wen-lu Zhang, Yu-wei Wang, Ai-long Huang, Jie-li Hu

**Affiliations:** ^1^Key Laboratory of Molecular Biology on Infectious Diseases, Ministry of Education, Chongqing Medical University, Chongqing, China; ^2^Department of Laboratory Medicine, Chongqing Hospital of Traditional Chinese Medicine, Chongqing, China

**Keywords:** hepatitis B virus, recombination, replication, RNA splicing, DNA sequence optimization

## Abstract

Recombinant DNA technology is a vital method in human hepatitis B virus (HBV), producing reporter viruses or vectors for gene transferring. Researchers have engineered several genes into the HBV genome for different purposes; however, a systematic analysis of recombinant strategy is lacking. Here, using a 500-bp deletion strategy, we scanned the HBV genome and identified two regions, region I (from nt 2,118 to 2,814) and region II (from nt 99 to 1,198), suitable for engineering. Ten exogenous genes, including puromycin N-acetyl transferase gene (*Pac*), blasticidin S deaminase gene (*BSD*), Neomycin-resistance gene (*Neo*), Gaussia luciferase (*Gluc*), NanoLuc (*Nluc*), *copGFP*, *mCherry*, *UnaG*, *eGFP*, and *tTA1*, were inserted into these two regions and fused into the open reading frames of hepatitis B core protein (HBC) and hepatitis B surface protein (HBS) via T2A peptide. Recombination of 9 of the 10 genes at region 99–1198 and 5 of the 10 genes at region 2118–2814 supported the formation of relaxed circular (RC) DNA. HBV DNA and HBV RNA assays implied that exogenous genes potentially abrogate RC DNA by inducing the formation of adverse secondary structures. This hypothesis was supported because sequence optimization of the UnaG gene based on HBC sequence rescued RC DNA formation. Findings from this study provide an informative basis and a valuable method for further constructing and optimizing recombinant HBV and imply that DNA sequence might be intrinsically a potential source of selective pressure in the evolution of HBV.

## Introduction

Infection with human hepatitis B virus (HBV) remains a public health problem around the world. More than 250 million people across the globe are estimated to be chronically infected with HBV (WHO, 2017; Polaris Observatory Collaborators, 2018; Schmit et al., 2020). Evidence shows that chronic HBV infection is a high-risk factor for the development of hepatocirrhosis and hepatocellular carcinoma.

**FIGURE 1:**
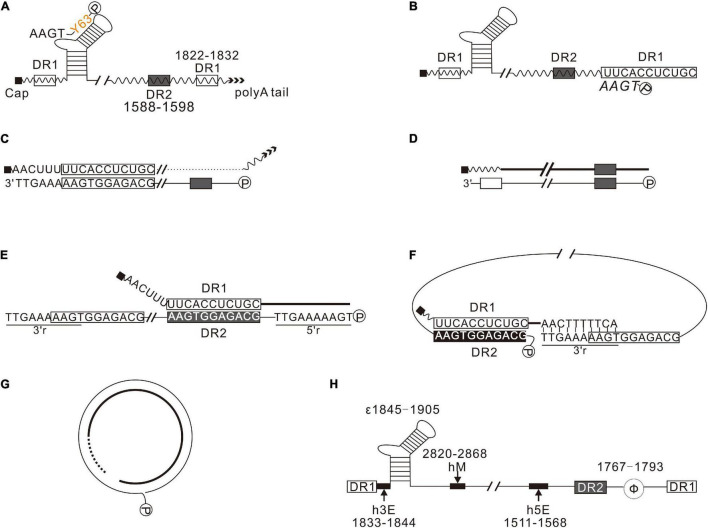
Synthesis of HBV DNA. **(A)** Pol (P) binds to 5′-proximal epsilon, forming a ribonucleoprotein complex with pgRNA, encapsidated into nucleocapsid formed by HBC. In the nucleocapsid, using the bulge of epsilon as a template, Pol synthesizes the minus-strand primer. **(B)** The nascent minus-strand primer translocates to 3′-proximal DR1 of pgRNA via base pairing. **(C)** pgRNA was degraded by the RNase H domain of Pol during reverse transcription, leaving a 11–16 nt long oligonucleotides, which serves as the primer for plus-strand synthesis. **(D)** A few plus-strand primers are retained *in situ* and extend to the 5′-end of minus-strand DNA, forming duplex linear DNAs (DL DNAs). **(E)** Most plus-strand primers translocate to DR2. Here, the synthesis of plus-strand resumes and extends to the 5′-end of minus-strand. **(F)** The nascent plus-strand pairs with the 3′r of minus-strand to complete circularization. **(G)** The plus-strand is synthesized further to form RC DNA. **(H)** *cis*-Elements play a crucial role in HBV replication. h5E (1511–1568), hM (2820–2868), and h3E (1833–1844) are crucial for plus-strand primer translocation. Φ (1767–1793) is important for minus-strand primer translocation.

HBV is a prototypical member of the Hepadnaviridae family, characterized by a relatively compact relaxed circular (RC) DNA genome and a special genomic replication mechanism via reverse transcription of a redundant RNA intermediate (Summers and Mason, 1982). The RC DNA genome of HBV is repaired after infection, converted into covalently closed circular DNA (cccDNA) in the nucleus of hepatocyte, and transcribed to pregenomic mRNA (pgRNA), precore mRNA, preS1 mRNA, S mRNA, and X mRNA. PgRNA functions as a bicistronic mRNA directing the synthesis of hepatitis B core protein (HBC) and polymerase (Pol) and is the template for reverse transcription. HBV replication begins with the encapsidation of pgRNA, whereby Pol binds to the stem-loop structure, epsilon (ε), at the 5′ end of pgRNA, triggering the assembly of HBC and packaging of the ribonucleoprotein complex into an icosahedral nucleocapsid (Bartenschlager et al., 1990; Bartenschlager and Schaller, 1992; Pollack and Ganem, 1993; Shin et al., 2002). The host chaperone proteins mediate encapsidation, and the nucleocapsid provides a microenvironment for reverse transcription (Shin et al., 2002). Briefly, the minus-strand primer, estimated at 3 or 4 nucleotides (nt) long, is synthesized in a protein primed process with the bulge of epsilon as a template. The minus-strand primer links to the tyrosine residue at the 63rd amino acid of Pol (Pol 63Y) via a covalent bond (Figure 1A; Nassal and Rieger, 1996; Lanford et al., 1997) and translocates to the complementary region, DR1, at the 3′ end of pgRNA (Figure 1B; Rieger and Nassal, 1996), where the synthesis of minus-strand DNA resumes. Subsequently, pgRNA is reverse transcribed into minus-strand and simultaneously degraded by the RNase H domain of Pol, leaving the 11–16 oligonucleotides at 5′-terminal of pgRNA undigested (Figure 1C; Summers and Mason, 1982). These oligonucleotides function as primers for plus-strand synthesis. A fraction of plus-strand primers retained *in situ* extends to the 5′ end of the minus-strand DNA and forms duplex linear DNAs (DL DNAs) (Figure 1D). Most plus-strand primers translocate to DR2 and extend to the 5′ end of the minus-strand, copying a 10-nt redundant sequence named 5′r (Figure 1E; Haines and Loeb, 2007). Subsequently, the nascent 3′ end of plus-strand pairs with the 3′-proximal redundant region (3′r) of minus-strand, continuing elongation of plus-strand to form RC DNA (Figures 1F,G).

*cis*-Elements, including epsilon (ε), direct repeat sequences (DR1 and DR2), terminal redundancy (5′ r and 3′r), h5E, hM, h3E, and Φ, are essential in every step of HBV replication (Figure 1H). Epsilon (ε) is the encapsidation signal and the template for protein priming (Pollack and Ganem, 1993); DR2 (1588–1598) and DR1 (1822–1832) are essential for primer translocations; 5′ r and 3′r, located at 1816–1824 of the minus-strand, are essential for circularization; h5E (1511–1568), hM (2820–2868), and h3E (1833–1844) play a critical role in plus-strand primer translocation (Liu et al., 2004; Lewellyn and Loeb, 2007); Φ (1767–1793) is essential for minus-strand primer translocation (Tang and McLachlan, 2002; Abraham and Loeb, 2006).

Recombinant DNA technology is a powerful tool for research in the field of HBV. The last 30 years have seen attempts by researchers to reconstruct HBV for different purposes. Insertion of differently sized exogenous genes, including HIV Tat, ZeoR, fluorescent proteins (GFP, EGFP, hrGFP, RFP, DsRed), Renilla luciferase (Rluc), Guassia luciferase (Gluc), NanoLuc luciferase (Nluc), and blasticidin S deaminase (BSD), into HBV genome has allowed for the construction of associated recombinant viruses (Kimura et al., 1994; Chaisomchit et al., 1997; Protzer et al., 1999; Yoo et al., 2002; Untergasser and Protzer, 2004; Liu et al., 2009, 2013; Hong et al., 2013; Nishitsuji et al., 2015, 2018). However, some of these efforts were unsuccessful (Bai et al., 2016), and no systematic analysis of HBV’s engineering strategy has been done.

In this view, we herein systematically analyzed the strategy of HBV engineering. First, a scan on the whole genome with a 500-bp deletion from position 1919 to position 1198 revealed two regions, nt 2,118–2,814 and nt 99–1,198, well-tolerable to deletion. Second, 10 exogenous genes, including puromycin N-acetyl transferase gene (*Pac*) (Lacalle et al., 1989), blasticidin S deaminase gene (*BSD*) (Kimura et al., 1994), Neomycin resistance gene (*Neo*) (Southern and Berg, 1982), Guassia luciferase (*Gluc*) (Verhaegent and Christopoulos, 2002), NanoLuc luciferase (*NLuc*) (England et al., 2016), *copGFP* (Shagin et al., 2004), *mCherry* (Shaner et al., 2004), *UnaG* (Kumagai et al., 2013), *eGFP* (Cormack et al., 1996), and *tTA1* (Baron et al., 1997), were inserted at position 2120 and position 155, respectively, via Thosea asigna virus 2A peptide (T2A) (Wang et al., 2015). Insertion of a majority of these genes supported RC DNA formation. We also systematically analyzed HBV RNA transcribed from different constructs to provide insight into how engineering affects the formation of RC DNA. The results showed that pgRNA splicing is common among those deletion variants and recombinants. However, this can hardly explain the failure of RC DNA formation of some recombinants. We thus provided a hypothesis that secondary structures induced by some of these exogenous genes might be potentially associated with abolishing RC DNA. This hypothesis was supported because sequence optimization of the UnaG gene based on HBC sequence, which is predicted to relieve the non-optimized UnaG on the structure of minus-strand DNA, rescued RC DNA formation.

## Materials and Methods

### Constructs

HBV DNA was derived from HBV subtype ayw (GenBank accession number v01460) and numbered according to the only *Eco*RI site of the genome. The “C” of the *Eco*RI site (GAATTC) was designated as position 1. Pch9/3091, constructed by Nassal et al., transcribes pgRNA under the control of the cytomegalovirus immediate-early promoter (pCMV) (Nassal, 1992). All HBV variants were constructed based on Pch9/3091 using Golden Gate Assembly with a few modifications (Engler et al., 2009). Briefly, the Golden Gate Assembly system comprised the following components: 1 × NEB buffer 3.1 (NEB), 5 mM DTT, 1 mM ATP, 0.5 units/μL of *Bsm*BI, or *Bsm*BI-v2 (NEB), 150 units/μL of T7 DNA Ligase (NEB), and 3–5 ng/μL of each DNA fragment. The reaction was performed with 60 cycles of 37°C for 5 min and 16°C for 5 min, followed by a 5-min incubation at 60°C. Plasmid pch9-G2016T contains a G2016T mutation, which terminates HBC translation at the 40th amino acid (HBC 40E). Pch9-Δε has a deletion between nt 1,858 and 1,863 and two substitutions (G1877T and T1878A). PgRNA transcribed from this construct would not be encapsidated but be translated to both HBC and Pol.

### Cell Culture and Transfection

HepG2 was cultured in DMEM/F12 supplemented with 10% fetal bovine serum and 10 μg/mL ciprofloxacin, and the culture medium was refreshed every 3 days (25–50% confluence) or every day (100% confluence). HepG2 cells were seeded into 12-well plates 20 h before transfection. Transfection was performed with Lipo8000 (Beyotime, China), following the manufacturer’s instructions.

### Core DNA Extraction and Southern Blotting

Plasmids were cotransfected, respectively, with pCH9-Δε, which expresses HBC and Pol *in trans*. Cells were harvested on day 5 posttransfection and washed once with phosphate-buffered saline (PBS). Core DNA was extracted as described previously (Abraham and Loeb, 2006). Briefly, cells in each well were lysed with 200 μL lysis buffer [50 mM Tris-HCl (pH 8.0), 1 mM EDTA, 0.2% NP40] and incubated at 37°C for 10 min. Lysed cells were centrifuged at 12,000 g for 5 min to remove the nuclei. The supernatant was supplemented with 5 mM CaCl_2_ and 5,000 units/mL micrococcal nuclease (NEB) and incubated at 37°C for 1.5 h. Subsequently, the micrococcal nuclease was inactivated with 10 mM EDTA. The reaction systems were supplemented with 0.5% sodium dodecyl sulfate and 0.5 mg/mL pronase and incubated at 37°C for 1.5 h. Core DNA was extracted with phenol–chloroform precipitated with ethanol and dissolved into 20 μL 1 × *Eco*RI buffer. Core DNA (10 μL) was digested by *Eco*RI for 1 h and resolved with 10 μL undigested samples on 1.5% agarose gel at 4 V/cm for 1.5 h. Following electrophoresis, the gel was denatured by soaking into two volumes of 0.4 M NaOH for 15 min with gentle shaking and transferred onto a positively charged nylon membrane with 60 mL 0.2 M NaOH overnight. The membranes were neutralized by soaking into 100 mL neutralization buffer (2 × saline- sodium phosphate- EDTA (SSPE), 200 mM Tris-HCl, pH 7.5) for 15 min with shaking and then UV-crosslinked at 1,500 × 100 μJ/cm^2^. Hybridization was performed via the DIG Easy Hyb (Roche, Germany) according to the manufacturer’s protocol using a digoxin-labelled probe (nt 1,199–1,814) at 30 ng/mL and 46°C. Detection of digoxin was performed following the manufacturer’s protocol (Roche, Germany), with some modifications according to a previously described method (Engler-Blum et al., 1993; McCabe et al., 1997). The NaCl concentration and the pH of the washing buffer were increased to 3 M and 8.0–9.0, respectively. In addition, 10 × blocking reagent was centrifuged at 13,000 g for 5 min, and the supernatant was added into washing buffer to reduce background.

### Total RNA Extraction and Northern Blotting

Total RNA was extracted using the TIANGEN RNA extraction reagent (TIANGEN, China) with some modifications. Briefly, cells were harvested on day 2 posttransfection and washed once with PBS. Next, cells were lysed in 500 μL/well Buffer RZ, and the lysate was supplemented with 1/5 volume of chloroform. The mixture was centrifuged at 13,000 g at 4°C for 10 min, and the supernatant was transferred into a new tube. Following phenol–chloroform extraction, total RNA was precipitated using ethanol and dissolved into 20 μL 1 × RNA loading buffer (60–73% formamide deioned, 1 × DNA loading buffer, and 10 μg/mL EtBr). RNA samples can be stored at -20°C for at least 1 month without degradation.

RNA samples were electrophoresed in TAE agarose gels according to a previously described method (Masek et al., 2005). The samples were first denatured at 65°C for 5 min and chilled on ice for 5 min before loading. Then, the samples were resolved on 1.2% 1 × TAE agarose gels at 4 V/cm and visualized under UV to examine the rRNAs. The gels were soaked into 120 mL 20 × saline sodium citrate (SSC) for at least 20 min. After that, RNA on the gels was transferred onto positively charged nylon membranes using 20 × SSC overnight. The membranes were UV-crosslinked directly on the next day. Hybridization and detection were performed by a similar method as the Southern blotting procedure except that the hybridization temperature was 60°C, and the low stringent washing temperature was 70°C.

### Reverse Transcription and Polymerase Chain Reaction

Reverse transcription was performed with TIANGEN FastKing RT Kit (TIANGEN, China) according to the manufacturer’s instructions with modifications. The incubation time was extended to 10 min in the DNA removal step and 30 min in the reverse transcription step. The reverse transcription primers included R HBV 1370, R HBV 1547, R HBV 1680, and R HBV 1800. Polymerase chain reaction (PCR) was performed with PrimeStar Max Premix (Takara, Japan) according to the manufacturer’s protocol.

### Sequence Optimization and DNA Structure Prediction

Sequence optimization of the exogenous genes was performed with DNAMAN version 8, using HBC sequence as the reference. Briefly, the DNA sequence of HBC was translated into protein, and the two most frequently used codons were used as the reference to optimize the sequence of selected genes. For DNA structure prediction, the secondary structure of DNA is predicted via DNAMAN version 8 as follows: load the sequences into the channel; select “sequence” > “secondary structure” > “current sequence.”

### Luciferase Assay

Plasmids were transfected into HepG2 as described above. For the luciferase assay in medium, 10 μL culture medium was collected on day 2 posttransfection and added to 30 μL 1 × Gluc assay buffer [0.5 × coelenterazine (Promega) in PBS supplemented with 5 mM NaCl] (Tannous, 2009), or 30 μL 1 × Nluc assay buffer [0.25 × furimazine (Promega) in PBS supplemented with 0.1% bovine serum albumin] (Boute et al., 2016). Chemiluminescence was detected by using a GloMax^®^ 20/20 Luminometer (Promega). For the luciferase assay in cell lysate, cells in each well were lysed with 200 μL lysis buffer (50 mM Tris-HCl (pH 8.0), 1 mM EDTA, 0.2% NP40) and incubated at 37°C for 10 min. Lysed cells were centrifuged at 12,000 g for 5 min to remove the debris. The supernatants (10 μL) were analyzed as described above.

### Blot Quantification and Statistical Analysis

According to the instructions on the website^1^, Southern and Northern blots were quantified by ImageJ 1.53e. The relative intensity of RC DNA and that of pgRNA were analyzed via a two-tailed Student *t*-test. *p* < 0.05 was considered statistically significant. QQ PLOT was used to determine the frequency distribution of the data analyzed using GraphPad Prism 8 (GraphPad Software, United States).

## Results

### Scanning of Hepatitis B Virus Genome for Desirable Recombination Sites

**FIGURE 2:**
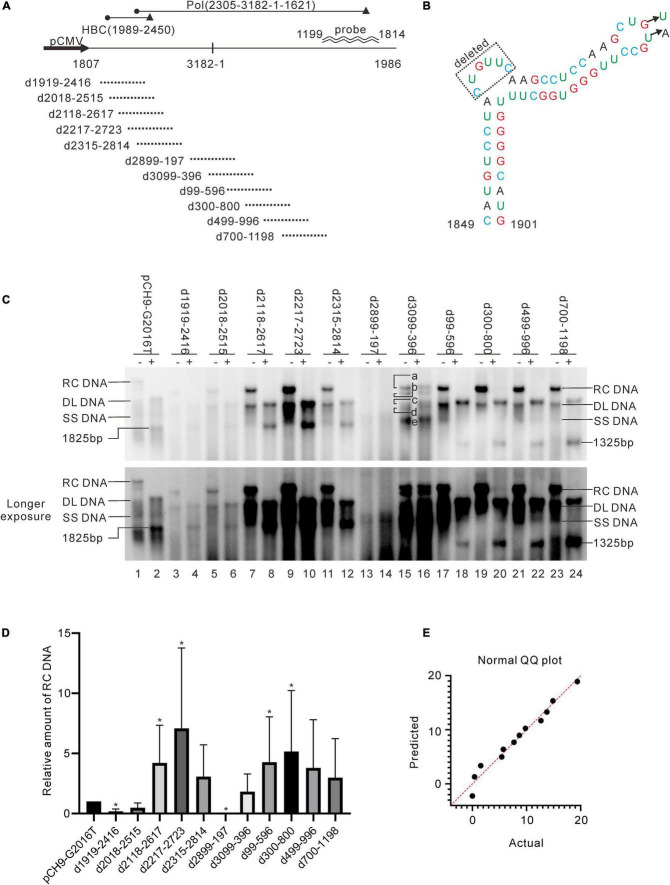
Identification of two regions suitable for engineering. **(A)** Sequences that were deleted in different constructs. Deletion variants were named according to the sequences deleted. The black cycle and black triangle represent the start codon and the stop codon, respectively. The hybridization probe is shown (1199–1814). **(B)** The mutations of pCH9-Δε. Sequence from 1858 to 1863 was deleted, and two additional mutations, G1877T and T1878A, were introduced. **(C)** Representative Southern blotting of the deletion variants. The plus sign (+) denotes *Eco*RI digestion. RC, DL, SS DNA, and *Eco*RI digested fragments are indicated, respectively. **(D)** The relative intensity of RC DNA of deletion variants. The intensity of RC DNA of each sample was divided into the sum of RC DNA of all samples in the same membrane, respectively, to calculate the relative amount of RC DNA. *N* = 5. Asterisks (*) denote the difference between a deletion variant and pCH9-G2016T is statistically significant (*p* < 0.05). **(E)** Normal QQ plot of the relative amount of the RC DNAs.

A series of deletion variants of the HBV genome was constructed based on Pch9/3091 to identify desirable regions for exogenous gene recombination (Figure 2A). Each variant had an approximated 500-bp deletion, spanning from 1919 to 1198 and skipping the *cis*-elements (Table 1). Pch9-Δε, in which the epsilon sequence was mutated to abort pgRNA encapsidation (Figure 2C), transcomplemented HBC and/or Pol for the damaged open reading frame (ORF) of HBC and/or Pol of some variants. The translation of HBC from Pch9-G2016T was terminated at the 40th amino acid by introducing a stop codon. Cotransfection of Pch9-G2016T and Pch9-Δε served as the positive control. All the variants were cotransfected with Pch9-Δε into HepG2. Core DNA was extracted on day 5 posttransfection and subjected to Southern blotting.

**TABLE 1:** Influence of deletion on the formation of RC DNA.

**Deletion region**	**Length of deletion (bp)**	**pgRNA splicing**	**Formation of RC DNA**
1919–2416	498	Nearly 100%	+, 7.0%
2018–2515	498	Nearly 100%	+, 26.9%
2118–2617	499	58%	+, 240.6%
2217–2723	507	50%	+, 338.9%
2315–2814	500	47%	+, 151.9%
2899–197	481	Nearly 100%	—
3099–396	480	74%	+, 94.8%
99–596	498	40%	+, 221.4%
300–800	501	17%	+, 260.2%
499–996	498	35%	+, 171.3%
700–1198	499	45%	+, 134.4%
2121–2819	699	ND	+, 45.9%

*ND, not determined.*

Pch9-G2016T formed three major bands, including RC, DL, and single-stranded (SS) DNA (Figure 2C, lane 1). The smear below SS DNA was either uncompleted intermediates of SS DNA or spliced products reverse transcribed from spliced pgRNA (Abraham et al., 2008). As expected, the RC DNA was linearized via *Eco*RI digestion, migrating to the same position as the undigested DL DNA. DL DNA was cut into two smaller linear fragments by *Eco*RI, with expected lengths of 1,365 bp (1814–3178) and 1,825 bp (1–1825), respectively, as 1814 is the transcription starting site of pgRNA from Pch9/3091 (Liu et al., 2004). Notably, the 1,365-bp fragment was not detected because the probe hybridizes with region 1199–1814. The 1,825-bp fragment migrated faster than SS DNA (Figure 2C, lane 2).

RC and DL DNA were detectable for all the variants except D2899-197 and D3099-396, which electrophoresed faster than their counterparts from Pch9-G2016T ascribed to a 500-bp deletion (Figure 2C). SS DNAs of these variants were not detected. Following *Eco*RI digestion, the RC DNAs of these variants migrated to the same position as the undigested DL DNA, providing evidence of their circular configuration. DL DNAs of D1919-2416, D2018-2515, D2118-2617, D2217-2723, and D2315-2814 were cut into two fragments, including one with an expected length of 1,825 bp and the other of 865 bp (nt 1,814–3,178 with a 500-bp deletion, undetectable). DL DNAs of D99-596, D300-800, D499-996, and D700-1198 were cut into two fragments, including one with a length of 1,325 bp (nt 1–1,825 with a 500-bp deletion) and the other with 1,365 bp (1814–3178, undetectable) (Figure 2C).

**FIGURE 3:**
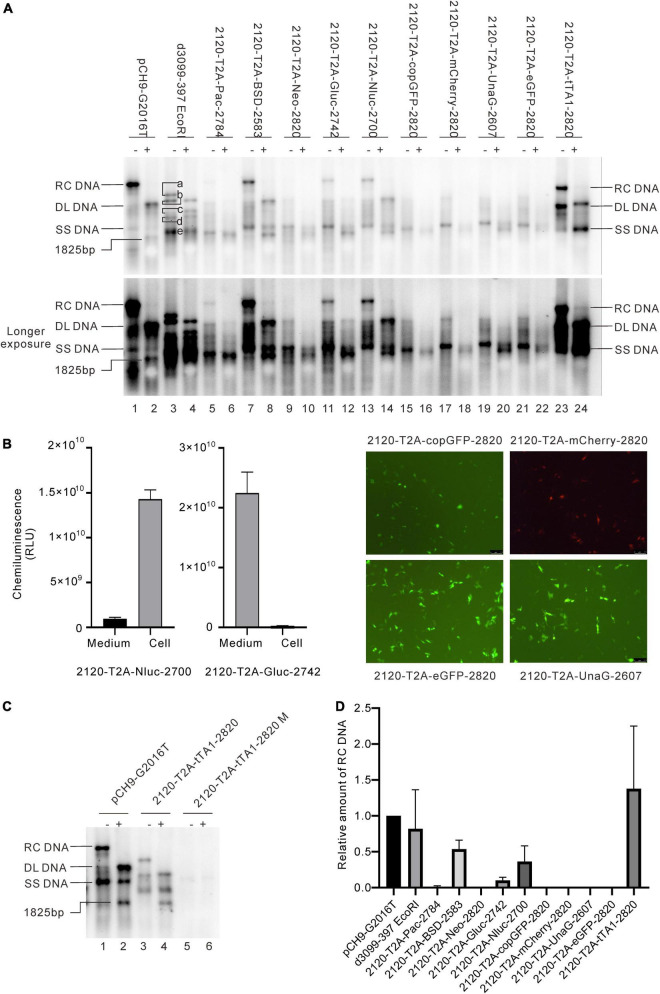
Influence of the recombination at 2120 on HBV DNA replication. **(A)** Representative Southern blotting results of the recombinants. The plus sign (+) denotes *Eco*RI digestion. RC, DL, SS DNA, and *Eco*RI digested fragments are shown. **(B)** Expression of the exogenous genes from the recombinants. Luciferase activity (Gluc and Nluc) or fluorescence (copGFP, eGFP, mCherry, and UnaG) was detected. **(C)** Representative Southern blotting of 2120-T2A-tTA1-2820 M. Notably, M denotes a deletion from nt 2064 to 2072 and a mutation in tetR (A426G). **(D)** The relative intensity of RC DNA of the recombinants. The intensity of each RC DNA was divided into the sum of RC DNA of all samples in the same membrane, respectively, to calculate the relative intensity of RC DNA of each sample. *N* = 4.

D3099-396 produced five bands (Figure 2C, lanes 15 and 16, bands a–e), all of which were resistant to *Eco*RI digestion because the *Eco*RI site was deleted (Figure 2A). Plasmid D3099-397*Eco*RI, in which an *Eco*RI site (GAATTC) was introduced between 3098 and 398, was constructed to verify whether D3099-396 formed RC DNA. Like D3099-396, five bands, indicated as a, b, c, d, and e, were detected in D3099-397*Eco*RI (Figure 3A, lane 3). Band “a” moved to the same position as band “c” after *Eco*RI digestion, while bands “b,” “d,” and “e” remained unchanged (Figure 3A, lanes 3 and 4). These results demonstrated that band “a” is RC DNA, band “c” is DL DNA, band “e” is SS DNA, and bands “b” and “d” are likely to be spliced products. Thus, D2899-197 is the only variant that does not support the formation of RC DNA.

### Deletions Associated With Hepatitis B Virus RNA Splicing

**FIGURE 4:**
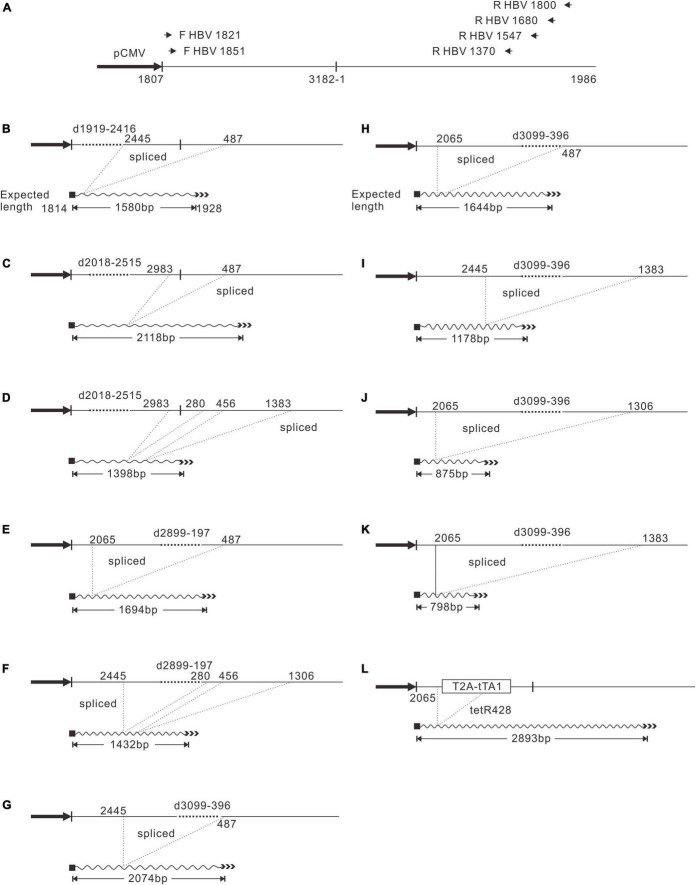
PgRNA splicing pattern of D1919-2416, D2018-2515, D2899-197, D3099-396, and 2120-T2A-tTA1-2820. **(A)** Primers used for reverse transcription and PCR. Each primer was named by the position of 5′ end, respectively. PgRNA was reverse transcribed with reverse primers and subjected to PCR amplification using each forward primer combined with each reverse primer. The shortest bands were gel-purified and sequenced. **(B)** PgRNA splicing of D1919-2416. **(C,D)** PgRNA splicing of D2018-2515. **(E,F)** PgRNA splicing of D2899-197. **(G–K)** PgRNA splicing of D3099-396. **(L)** PgRNA splicing of 2120-T2A-tTA1-2820.

It is intriguing that D2899-197 formed only weak SS DNA (Figure 2C, lanes 13 and 14) without impairing known *cis*-elements. One hypothesis is that the pgRNA expressed from this construct was spliced (Abraham et al., 2008). To address this question, we performed a systematic analysis of the influence of the deletions on HBV RNA splicing. Total RNA samples were assayed by Northern blotting and reverse transcribed into cDNA using primers R HBV 1370, R HBV 1547, R HBV 1680, and R HBV 1800, respectively, to identify whether the pgRNAs were spliced (Figure 4A). The cDNA was further amplified via PCR using primers F HBV 1821 or F HBV 1851 plus R HBV 1370, or R HBV 1547, or R HBV 1680, or R HBV 1800, respectively, and PCR products were gel-purified and sequenced.

**FIGURE 5:**
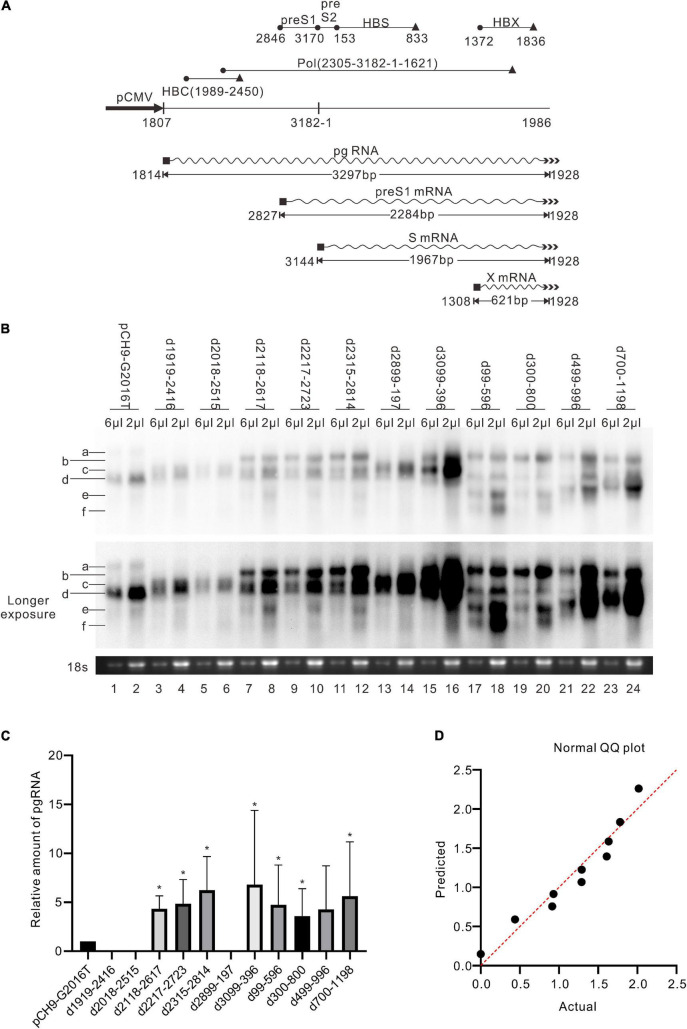
RNA assay of the deletion variants. **(A)** The sequence of HBV transcripts. The wave lines represent RNA; the black rectangles represent 5′ cap; the multiple arrowheads represent the poly-A tail. The black cycles and black triangles represent the start codon and the stop codon, respectively. The length of each transcript is an estimation without a poly-A tail. **(B)** Representative Northern blotting results of the deletion variants. **(C)** The relative intensity of pgRNA of the deletion variants. The intensity of each pgRNA/18s rRNA was divided into the sum of pgRNA/18s rRNA of all samples in the same membrane, respectively, to calculate the relative intensity of pgRNA of each sample. *N* = 4. Asterisks (*) denote the difference between deletion variants and pCH9-G2016T is significant (*p* < 0.05). **(D)** Normal QQ plot of the relative amount of the pgRNAs.

Pch9-G2016T, as the positive control, is expected to transcribe pgRNA, preS1 mRNA, S mRNA, and X mRNA, regulated by cytomegalovirus immediate-early promoter (pCMV), S1 promoter, S2 promoter, and X promoter, respectively (Figure 5A; Panjaworayan et al., 2007). Notably, these RNAs presented as three major bands in Northern blotting (Figure 5B). Band “a” was pgRNA, and band “e” was X mRNA. Band “d,” possibly comprised two close bands, was suggested to be preS1 and S mRNAs. Deletion variants D2118-2617, D2217-2723, D2315-2814, D3099-396, D99-596, D300-800, D499-996, and D700-1198 produced shorter pgRNAs than that of the control, owing to a 500-bp deletion for each (band “b,” Figure 5B, lanes 7–12 and 15–24). However, variants D2899-197 showed no bands at position “b,” but rather a smear at position “c.” HBV RNA of this variant was reverse transcribed, and the corresponding DNA fragments were amplified and sequenced as described previously. Indeed, the sequencing results demonstrated that the pgRNA of D2899-197 was spliced with two patterns; one is 2065-487 (i.e., the sequence between nt 2065 and 487 is missing), and the other is spliced twice, from nt 2,445 to 280 and from nt 456 to 1,306 (Figures 4E,F).

Moreover, splicing was detected in other deletion variants. D1919-2416 showed one splicing pattern, from nt 2,445 to 487 (Figure 4B). D2018-2515 had two splicing patterns: one is from nt 2,983 to 487, and the other is spliced twice, from nt 2,983 to 280, and from nt 456 to 1,383 (Figures 4C,D). However, the splicing rates of pgRNAs of these two variants were not 100% because the two variants did support the formation of intact RC DNA (Figure 2C, lanes 3–6), which must be derived from complete pgRNA. Notably, the pgRNA of D3099-396 was spliced to a less extent but via a more complex mechanism. At least five splicing patterns, from nt 2,445 to 487, from nt 2,065 to 487, from nt 2,445 to 1,383, from nt 2,065 to 1,306, and from nt 2,065 to 1,383, respectively, were revealed (Figures 4G–K). Some of these splicings are potential sources of the additional bands of D3099-396 detected via Southern blotting (“b” and “d” in Figure 2C, lane 15). Analysis of the splicing of pgRNA of D99-596, D300-800, D499-996, and D700-1198 is challenging because of the complicated bands.

In addition, the amount of pgRNA of deletion variants (except D1919-2416, D2018-2515, D2899-197, and D499-996) was significantly higher than Pch9-G2016T, respectively (*p* < 0.05) (Figure 5C). This observation provides evidence of these constructs’ relatively higher amount of RC DNA (Figures 2D, 5C).

### Deletions of 2,118–2,814 and 99–1,198 Produces More Relaxed Circular DNA Than Other Variants

Regions with the least impact on the formation of RC DNA were identified by comparing the relative amount of RC DNAs among these variants. Southern blotting was repeated five times, followed by analyzing the relative intensity of RC DNAs with ImageJ. Results demonstrated that the relative intensities of RC DNA of D2118-2617, D2217-2723, D99-596, and D300-800 were significantly higher than Pch9-G2016T (Figure 2D), whereas that of D1919-2416 and D2899-197 were significantly lower than Pch9-G2016T. Also, D2315-2814, D3099-396, D499-996, and D700-1198 produced a similar amount of RC DNA as Pch9-G2016T (Figure 2D). These results demonstrate that two regions are suitable for recombination, including 2,118–2,814 and 99–1,198.

### Influence of Recombination of Exogenous Genes at 2120 on Relaxed Circular DNA Formation

Given the data above, two positions, 2120 and 155, located in the ORF of HBC and HBS, respectively, were selected to insert foreign genes. First, three selection genes (Pac, BSD, and Neo), two luciferase genes (Gluc and Nluc), four fluorescent genes (copGFP, mCherry, UnaG, and eGFP), and one transactivating gene (tTA1) were inserted right after the valine residue at the 74th amino acid of HBC (HBC 74V, site 2120) (Table 2). Each gene was fused via a T2A peptide at the N-terminal to separate its expression from the HBV genome. The focus was on the formation of RC DNA as it is the precursor of functional cccDNA.

**TABLE 2:** Influence of exogenous gene engineering on the formation of RC DNA.

**Location**	**Exogenous gene**	**Length of gene (bp)**	**HBV region replaced**	**Expected change of pgRNA length (bp)**	**pgRNA splicing**	**Formation of RC DNA**
Site 2120 (HBC and Pol region)	T2A-Pac	663	2121–2783	0	++	Weak, 1.3%
	T2A-BSD	462	2121–2582	0	+	+, 52.9%
	T2A-Neo	858	2121–2819	+159	++	–
	T2A-Gluc	621	2121–2741	0	+	+, 10.2%
	T2A-Nluc	579	2121–2699	0	++	+, 33.8%
	T2A-copGFP	723	2121–2819	+24	++	–
	T2A-mCherry	774	2121–2819	+75	++	–
	T2A-UnaG	486	2121–2606	0	++	–
	T2A-eGFP	784	2121–2819	+83	+	–
	T2A-tTA1	840	2121–2819	+140	++++	Spliced, 117.3%
Site 155 (HBS region)	T2A-Pac	663	156–518	0	+	Weak, 5.6%
	T2A-BSD	462	156–617	0	+	+, 64.4%
	T2A-Neo	858	156–1013	0	+	+, 33.8%
	T2A-Gluc	621	156–776	0	+	+, 76.2%
	T2A-Nluc	579	156–734	0	+	+, 79.7%
	T2A-copGFP	723	156–878	0	+	+, 18.9%
	T2A-mCherry	774	156–929	0	+	–
	T2A-UnaG	486	156–641	0	++	+, 33.9%
	T2A-eGFP	784	156–938	0	+	+, 25.0%
	T2A-tTA1	840	156–994	0	+	+, 25.5%

*++++, > 75%; +++, 50%−75%; ++, 25%−50%; +, 10%−25%.*

Results revealed that 2120-T2A-Pac-2784, 2120-T2A-BSD-2583, 2120-T2A-Gluc-2742, and 2120-T2A-Nluc-2700 formed RC DNA, migrating similarly to that of Pch9-G2016T (Figure 3A). RC DNAs were verified through *Eco*RI digestion, whereby the RC DNAs were linearized to the same position as the undigested DL DNAs (Figure 3A). The expression of exogenous genes was confirmed by the detection of chemiluminescence or fluorescence (Figure 3B). As for the amount of RC DNA, less of RC DNA of these constructs was reported than Pch9-G2016T. Insertion of BSD and Nluc maintained more RC DNAs than the insertion of Pac and Gluc (Figure 3D). 2120-T2A-tTA1-2820 formed an RC DNA band migrating faster than Pch9-G2016T, 2120-T2A-BSD-2583, 2120-T2A-Gluc-2742, and 2120-T2A-Nluc-2700 (Figure 3A, lanes 1, 2, 7, 8, 11–14, 23, and 24). It was speculated that the shorter product was potentially synthesized from spliced pgRNA.

A part of constructs formed only DL and SS DNA, including 2120-T2A-Neo-2820, 2120-T2A-copGFP-2820, 2120-T2A-mCherry-2820, 2120-T2A-UnaG-2607, and 2120-T2A-eGFP-2820 (Figure 3A). *Eco*RI digestion yielded 1,825-bp fragments, confirming the identity of DL DNAs. Notably, SS DNAs of 2120-T2A-copGFP-2820 and 2120-T2A-eGFP-2820 electrophoresed slightly faster than others (Figure 3A, lanes 15 and 21).

### Splicing of pgRNAs of the 2120 Recombinants

**FIGURE 6:**
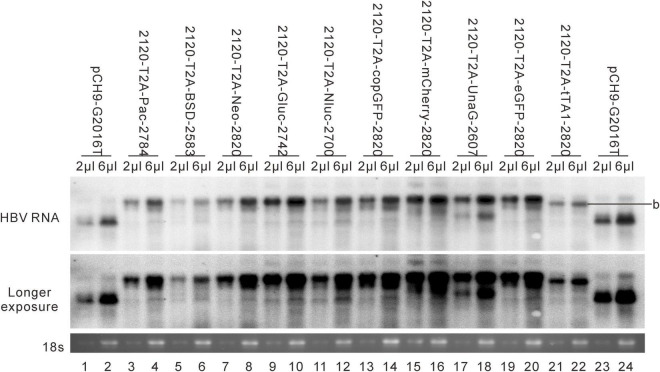
RNA assay for the 2120 recombinants. Representative Northern blotting is shown.

Different genes exert different effects on the formation of RC DNA. Therefore, to pursue the underlying reasons, we analyzed the RNAs from a part of the recombinants. All the recombinants tested, to some extent, showed pgRNA splicing (the bands right under pgRNA bands), although intact pgRNA was observed in all samples (Figure 6 and Table 2). The spliced pgRNAs possibly explain the small RC DNAs of 2120-T2A-BSD-2583, 2120-T2A-Gluc-2742, and 2120-T2A-Nluc-2700 (Figure 3A). However, for 2120-T2A-tTA1-2820, the pgRNA was spliced approximately 80% (band “b” Figure 6, lanes 21 and 22). Sequencing results demonstrated that this RNA was spliced between HBV nt 2,065 and tetR nt 428 (sequence from HBV nt 2,065 to nt 428 of tetR was missing) (Figure 4L), providing potential evidence on why the RC DNA of 2120-T2A-tTA1-2820 electrophoresed faster than the RC DNA of Pch9-G2016T (Figure 3A, lanes 1 and 23). The splicing sites of 2120-T2A-tTA1-2820 were removed by deleting nt 2,064–2,072 and mutating A426 of tetR to G426. However, this manipulation did not rescue RC DNA formation (Figure 3C, lanes 5 and 6).

### Influence of Recombination of Exogenous Genes at 155 on Relaxed Circular DNA Formation

**FIGURE 7:**
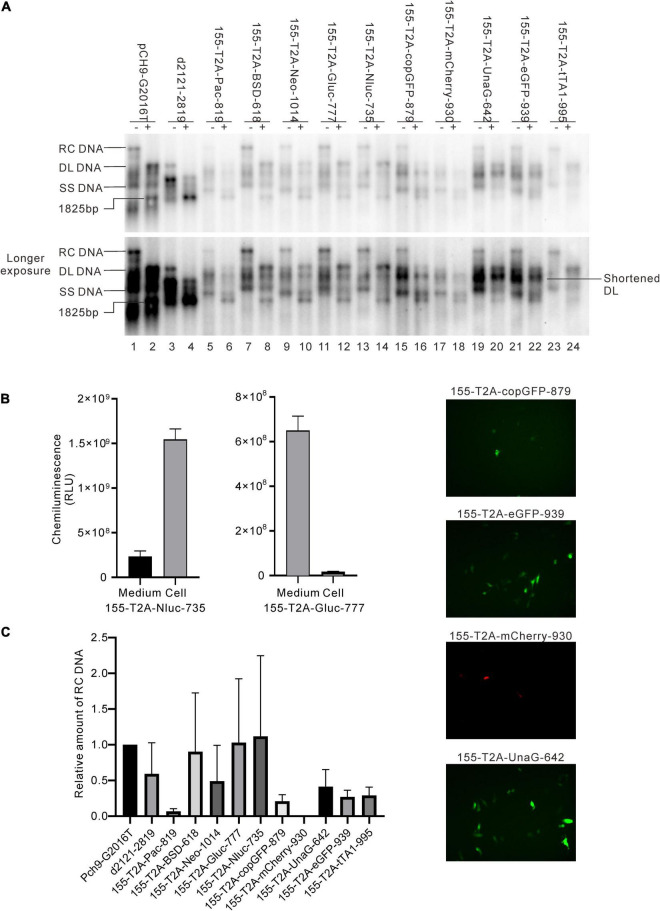
Influence of the recombination at 155 on RC DNA formation. **(A)** A representative Southern blotting is shown. The plus sign (+) denotes *Eco*RI digestion. RC, DL, SS DNA, and *Eco*RI digested fragments are indicated. **(B)** Expression of the exogenous genes from the recombinants. Luciferase activity (Gluc and Nluc) or fluorescence (copGFP, eGFP, mCherry, and UnaG) was detected. **(C)** The relative intensity of RC DNA of the recombinants. The intensity of RC DNA of each sample was divided into the sum of RC DNA of all samples in the same membrane, respectively, to calculate the relative intensity of RC DNA of each sample. *N* = 4.

**FIGURE 8:**
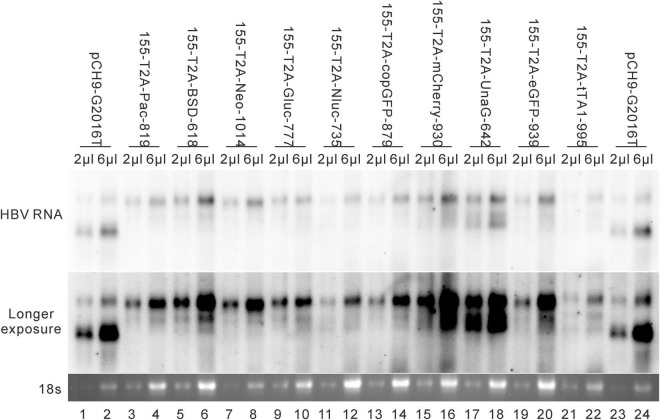
RNA assay for the 155 recombinants. Representative Northern blotting is shown.

These 10 exogenous genes were also inserted right after the first methionine residue of HBS (site 155). Except for 155-T2A-mCherry-930, all of these insertions supported RC formation (Figure 7A). All the RC DNAs electrophoresed at the same position as that of Pch9-G2016T. Following *Eco*RI digestion, the RC DNAs shifted to undigested DL DNAs, and DL DNAs moved to 1,825-bp fragments. The expression of exogenous genes was confirmed by the detection of chemiluminescence or fluorescence (Figure 7B). Regarding the amount of RC DNA, constructs with BSD, Gluc, and Nluc were higher than those with Neo, copGFP, UnaG, eGFP, tTA1, and Pac (Figure 7C). 155-T2A-mCherry-930 only formed DL and SS DNA (Figure 7A, lanes 17 and 18). However, RNA assay demonstrated that 155-T2A-mCherry-930 produced normal pgRNA (Figure 8), supporting that RC DNA formation failure is attributed to obstacles in DNA synthesis steps. Shortened DL bands from 155-T2A-Pac-819, 155-T2A-BSD-618, 155-T2A-Neo-1014, 155-T2A-Gluc-777, 155-T2A-copGFP-879, 155-T2A-UnaG-642, and 155-T2A-eGFP-939 could be reverse transcribed from spliced RNA (compare Figure 7A with Figure 8).

### Sequence Optimization Improves Relaxed Circular DNA Formation for Some Recombinant Constructs

**FIGURE 9:**
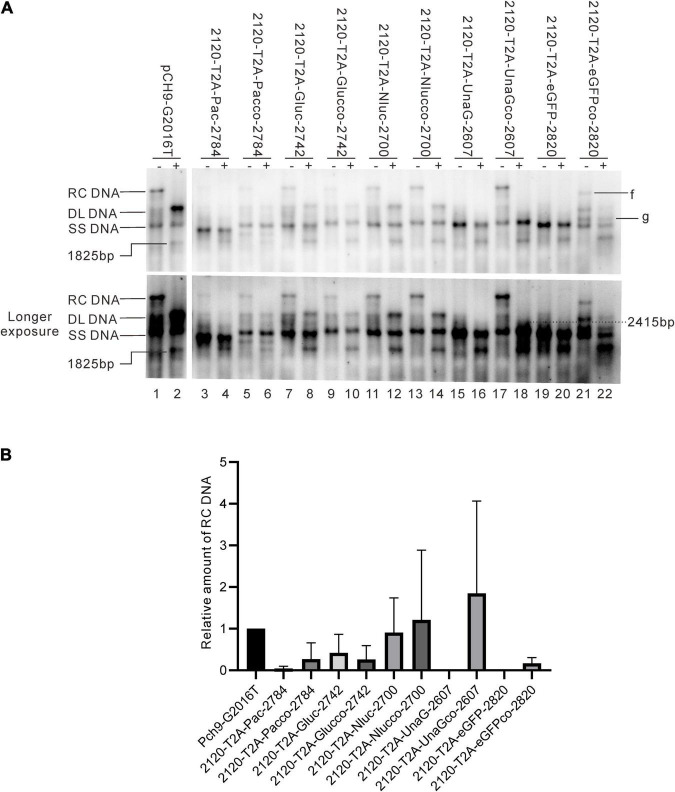
Influence of sequence optimization of exogenous genes on HBV replication. **(A)** A comparison of HBV DNA from the recombinants before and after sequence optimization. The plus sign (+) denotes *Eco*RI digestion. RC, DL, SS DNA, and *Eco*RI digested fragments are indicated. **(B)** The relative intensity of RC DNA. The intensity of each RC DNA was divided into the sum of RC DNA of all samples in the same membrane to calculate the relative intensity of RC DNA of each sample. *N* = 4.

**FIGURE 10:**
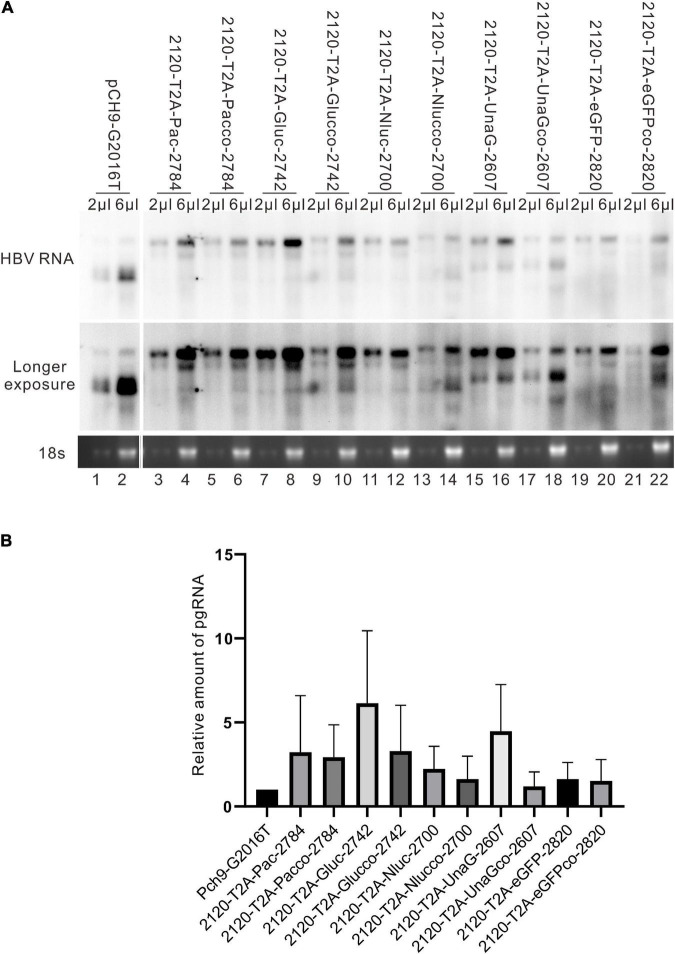
HBV RNA assay of the recombinants before and after sequence optimization. **(A)** Representative Northern blotting of the recombinants before and after sequence optimization. **(B)** The relative intensity of pgRNA of the recombinants before and after sequence optimization. The ratio of each pgRNA/18s rRNA was calculated, and this ratio was then divided into the sum of pgRNA/18s rRNA of all samples in the same membrane to calculate the relative intensity of pgRNA of each sample. *N* = 3.

**FIGURE 11:**
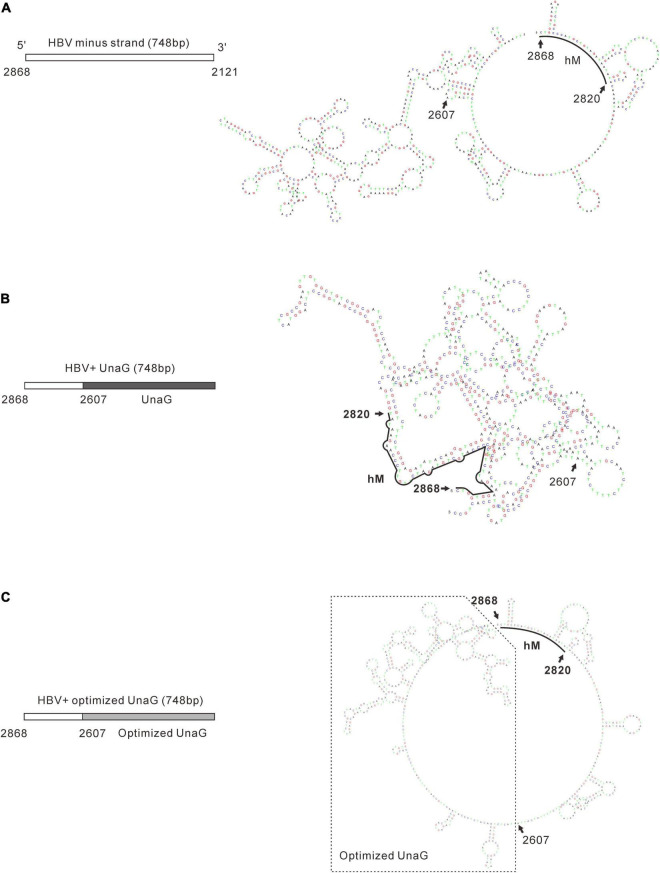
Prediction of the secondary structure of minus-strand DNA of the recombinants. Secondary structures of the minus-strand DNA of wild-type HBV **(A)**, unoptimized UnaG recombinant **(B)**, and optimized UnaG **(C)** were predicated by using DNAMAN 8. The structure of *cis*-element hM (nt 2820–2868) is indicated. It is obvious that unoptimized UnaG profoundly affects the structure of HBV sequence, including hM. In contrast, optimized UnaG showed no significant impacts on the structure of the HBV sequence (nt 2607–2868).

It was reported that insertion of UnaG aborted RC DNA formation at nt 2,120 but supported that at nt 155. Furthermore, recombination of UnaG maintained DL and SS DNA formation, suggesting the blockade of the translocation or circularization of the plus-strand primer, which is dependent on where the foreign genes were inserted. These results implied that the insertion of UnaG at nt 2,120 might form the undesirable secondary or higher-order structure of minus-strand DNA, which possibly affects either the translocation of the plus-strand primer or the circularization step. Guided by this hypothesis, we optimized the sequence of genes inserted according to the DNA sequence of HBC, expecting that a DNA sequence resembling HBC would reduce the influence on replication. Five genes (*UnaG*, *Pac*, *Gluc*, *Nluc*, and *eGFP*) were optimized (Table 3) and inserted into the same positions as the unoptimized genes, respectively. Of note, the construct with optimized UnaG (UnaGco) formed RC DNA in a similar amount as that of the positive control (Pch9-G2016T). This band migrated to the same position as the RC DNA of Pch9-G2016T (Figure 9A, lanes 17 and 18). *Eco*RI digestion generated a fragment that electrophoresed faster than undigested DL DNA but slower than SS DNA of 2120-T2A-UnaGco-2607 (Figure 9A, lanes 17 and 18). These findings affirmed RC DNA identity because there is an *Eco*RI recognition site in UnaGco. *Eco*RI digestion is expected to produce a detectable band of 2,145 bp (the other should be 759 bp undetectable by our probe). In contrast, 2120-T2A-UnaG-2607 formed only DL and SS DNA (Figure 9A, lanes 15 and 16). The weak DL DNA could be revealed solely via *Eco*RI digestion, which cut DL DNA to a detectable 1,825-bp fragment. Northern blotting assay of HBV RNA demonstrated a significantly lower relative amount of intact pgRNA of 2120-T2A-UnaGco-2607 than that of 2120-T2A-UnaG-2607, whereas a part of pgRNA of both could be spliced (Figures 10A,B) indicating that the improvement in RC DNA formation by sequence optimization must be explained by improvement in the steps after pgRNA production. To address this, we analyzed the secondary structure of the sequence from the minus-strand DNAs by using DNAMAN 8. Three sequences corresponding to wild type HBV (nt 2,121–2,868), chimeric UnaG-HBV, and optimized UnaG-HBV (Figure 11) were predicted for secondary structure. Wild-type UnaG sequence profoundly impacts the overall structure of HBV (Figures 11A,B). Especially, the structure of *cis*-element hM is different between these two. On the contrary, optimized UnaG does not significantly influence the structure of HBV DNA fused (Figure 11C), with the hM showing a similar structure as that of wild-type HBV DNA. The influence of sequence optimization of Pac (Pacco) on HBV DNA replication seemed different. The SS DNA of 2120-T2A-Pac-2784 electrophoresed faster than the SS DNA of D3099-397 *Eco*RI (Figure 3A, lanes 3 and 5), demonstrating that the SS DNA of 2120-T2A-Pac-2784 was at least 487 bp shorter than the SS DNA of Pch9-G2016T. Evidence suggests that the shorter SS DNA is possibly an immature product paused by assumptive secondary RNA structures formed by the Pac gene, with a high content of “G and C” (72.8%). In line with this hypothesis, optimized Pac (Pacco, 2120-T2A-Pacco-2784) promoted SS DNA maturation (Figure 9A, lanes 3–6). The RC DNA intensity was only slightly enhanced without significance by Pac optimization (*p* > 0.05). 2120-T2A-Glucco-2742 and 2120-T2A-Nlucco-2700 formed RC, DL, and SS DNA (Figure 9A, lanes 7–14). At the same time, sequence optimization of Nluc and Gluc did not augment the amount of RC DNA further. Also, the pgRNA amount of 2120-T2A-Glucco-2742 and 2120-T2A-Nlucco-2700 were lower than the unoptimized counterparts (Figure 10).

**TABLE 3:** The sequence of exogenous genes before and after optimization.

**Genes**	**The sequence of exogenous genes before and after optimization**						
Pac	1	ATGACCGAGT	ACAAGCCCAC	GGTGCGCCTC	GCCACCCGCG	ACGACGTCCC	CAGGGCCGTA
	61	CGCACCCTCG	CCGCCGCGTT	CGCCGACTAC	CCCGCCACGC	GCCACACCGT	CGATCCGGAC
	121	CGCCACATCG	AGCGGGTCAC	CGAGCTGCAA	GAACTCTTCC	TCACGCGCGT	CGGGCTCGAC
	181	ATCGGCAAGG	TGTGGGTCGC	GGACGACGGC	GCCGCGGTGG	CGGTCTGGAC	CACGCCGGAG
	241	AGCGTCGAAG	CGGGGGCGGT	GTTCGCCGAG	ATCGGCCCGC	GCATGGCCGA	GTTGAGCGGT
	301	TCCCGGCTGG	CCGCGCAGCA	ACAGATGGAA	GGCCTCCTGG	CGCCGCACCG	GCCCAAGGAG
	361	CCCGCGTGGT	TCCTGGCCAC	CGTCGGCGTC	TCGCCCGACC	ACCAGGGCAA	GGGTCTGGGC
	421	AGCGCCGTCG	TGCTCCCCGG	AGTGGAGGCG	GCCGAGCGCG	CCGGGGTGCC	CGCCTTCCTG
	481	GAGACCTCCG	CGCCCCACAA	CCTCCCCTTC	TACGAGCGGC	TCGGCTTCAC	CGTCACCGCC
	541	GACGTCGAGG	TGCCCGAAGG	ACCGCGCACC	TGGTGCATGA	CCCGCAAGCC	CGGTGCCTGA
Pacco (optimized)	1	ATGACTGAAT	ATAAACCTAC	CGTTAGACTA	GCTACACGAG	ATGATGTGCC	AAGGGCCGTA
	61	AGAACTCTCG	CTGCCGCTTT	TGCCGACTAC	CCTGCTACCC	GACATACAGT	CGATCCAGAC
	121	AGGCACATTG	AGAGAGTTAC	TGAATTGCAA	GAGCTATTCC	TCACCCGAGT	GGGATTGGAT
	181	ATCGGCAAGG	TATGGGTCGC	CGACGATGGA	GCTGCCGTTG	CTGTGTGGAC	AACTCCTGAA
	241	TCTGTAGAGG	CCGGCGCTGT	CTTTGCCGAA	ATTGGACCAA	GGATGGCTGA	GCTATCAGGC
	301	TCTAGACTCG	CCGCTCAGCA	ACAGATGGAA	GGATTGCTAG	CCCCTCATCG	ACCAAAAGAG
	361	CCTGCTTGGT	TCCTCGCCAC	CGTTGGCGTG	TCACCAGACC	ACCAAGGAAA	GGGCTTGGGA
	421	TCTGCTGTAG	TCCTACCTGG	CGTTGAAGCC	GCTGAGAGGG	CCGGAGTGCC	AGCTTTTCTC
	481	GAAACATCAG	CCCCTAGAAA	TTTGCCATTC	TATGAGCGAC	TAGGCTTTAC	TGTAACCGCT
	541	GATGTCGAAG	TTCCTGAGGG	ACCAAGGACA	TGGTGTATGA	CTAGAAAACC	TGGCGCCTGA
Gluc	1	ATGGGAGTCA	AAGTTCTGTT	TGCCCTGATC	TGCATCGCTG	TGGCCGAGGC	CAAGCCCACC
	61	GAGAACAACG	AAGACTTCAA	CATCGTGGCC	GTGGCCAGCA	ACTTCGCGAC	CACGGATCTC
	121	GATGCTGACC	GCGGGAAGTT	GCCCGGCAAG	AAGCTGCCGC	TGGAGGTGCT	CAAAGAGATG
	181	GAAGCCAATG	CCCGGAAAGC	TGGCTGCACC	AGGGGCTGTC	TGATCTGCCT	GTCCCACATC
	241	AAGTGCACGC	CCAAGATGAA	GAAGTTCATC	CCAGGACGCT	GCCACACCTA	CGAAGGCGAC
	301	AAAGAGTCCG	CACAGGGCGG	CATAGGCGAG	GCGATCGTCG	ACATTCCTGA	GATTCCTGGG
	361	TTCAAGGACT	TGGAGCCCAT	GGAGCAGTTC	ATCGCACAGG	TCGATCTGTG	TGTGGACTGC
	421	ACAACTGGCT	GCCTCAAAGG	GCTTGCCAAC	GTGCAGTGTT	CTGACCTGCT	CAAGAAGTGG
	481	CTGCCGCAAC	GCTGTGCGAC	CTTTGCCAGC	AAGATCCAGG	GCCAGGTGGA	CAAGATCAAG
	541	GGGGCCGGTG	GTGACTAA				
Glucco (optimized)	1	ATGGGCGTGA	AAGTTCTCTT	CGCTCTCATC	TGCATCGCTG	TTGCCGAAGC	TAAGCCGACA
	61	GAGAATAATG	AAGATTTTAA	TATTGTTGCT	GTCGCCTCAA	ATTTCGCTAC	TACTGATCTA
	121	GACGCTGATA	GAGGTAAATT	GCCGGGCAAA	AAGCTTCCTC	TCGAGGTCCT	CAAAGAAATG
	181	GAGGCAAATG	CCAGGAAAGC	TGGCTGTACT	AGGGGATGTT	TGATATGTCT	ATCTCATATA
	241	AAATGCACTC	CAAAAATGAA	AAAGTTCATT	CCAGGTCGAT	GTCATACATA	TGAAGGAGAC
	301	AAAGAATCTG	CCCAAGGAGG	TATTGGAGAA	GCGATTGTGG	ACATACCAGA	AATTCCTGGA
	361	TTTAAGGATT	TGGAACCAAT	GGAACAATTT	ATTGCTCAAG	TAGATCTATG	TGTGGACTGT
	421	ACTACTGGAT	GTTTGAAGGG	CCTAGCCAAT	GTACAATGTA	GTGACCTCTT	AAAAAAATGG
	481	CTTCCTCAAA	GATGTGCTAC	TTTTGCATCA	AAAATTCAAG	GCCAAGTAGA	TAAAATTAAA
	541	GGCGCTGGCG	GTGACTAA				
Nluc	1	ATGGTCTTCA	CACTCGAAGA	TTTCGTTGGG	GACTGGCGAC	AGACAGCCGG	CTACAACCTG
	61	GACCAAGTCC	TTGAACAGGG	AGGTGTGTCC	AGTTTGTTTC	AGAATCTCGG	GGTGTCCGTA
	121	ACTCCGATCC	AAAGGATTGT	CCTGAGCGGT	GAAAATGGGC	TGAAGATCGA	CATCCATGTC
	181	ATCATCCCGT	ATGAAGGTCT	GAGCGGCGAC	CAAATGGGCC	AGATCGAAAA	AATTTTTAAG
	241	GTGGTGTACC	CTGTGGATGA	TCATCACTTT	AAGGTGATCC	TGCACTATGG	CACACTGGTA
	301	ATCGACGGGG	TTACGCCGAA	CATGATCGAC	TATTTCGGAC	GGCCGTATGA	AGGCATCGCC
	361	GTGTTCGACG	GCAAAAAGAT	CACTGTAACA	GGGACCCTGT	GGAACGGCAA	CAAAATTATC
	421	GACGAGCGCC	TGATCAACCC	CGACGGCTCC	CTGCTGTTCC	GAGTAACCAT	CAACGGAGTG
	481	ACCGGCTGGC	GGCTGTGCGA	ACGCATTCTG	GCGTAA		
Nlucco (optimized)	1	ATGGTTTTTA	CTCTCGAAGA	TTTTGTTGGT	GACTGGAGAC	AAACTGCTGG	ATATAATCTA
	61	GATCAAGTAT	TAGAACAAGG	AGGTGTATCA	TCTTTATTTC	AAAATTTAGG	AGTATCGGTA
	121	ACTCCTATTC	AAAGAATCGT	TCTTTCTGGC	GAAAATGGTT	TGAAAATCGA	CATTCATGTA
	181	ATCATTCCCT	ATGAAGGACT	CTCAGGAGAT	CAAATGGGAC	AAATTGAAAA	AATTTTTAAA
	241	GTGGTTTATC	CTGTAGATGA	TCACCATTTT	AAAGTCATCC	TACATTATGG	AACTTTAGTG
	301	ATAGATGGTG	TGACTCCTAA	TATGATCGAC	TATTTCGGAA	GACCTTATGA	AGGAATTGCT
	361	GTATTTGACG	GCAAGAAAAT	TACTGTTACT	GGTACTCTGT	GGAATGGAAA	TAAAATTATC
	421	GATGAGCGGT	TAATCAACCC	TGATGGATCA	CTACTTTTCC	GAGTTACCAT	TAATGGGGTT
	481	ACCGGTTGGA	GACTCTGTGA	ACGCATTCTA	GCGTAA		
UnaG	1	ATGATCCTGG	AAAAATTCGT	CGGCACTTGG	AAGATCGCCG	ACAGCCACAA	CTTCGGCGAG
	61	TACCTGAAGG	CCATCGGCGC	CCCCAAGGAG	CTGTCTGACG	GCGGCGACGC	CACCACTCCC
	121	ACCCTGTATA	TCTCCCAGAA	AGACGGCGAC	AAGATGACCG	TGAAGATCGA	GAACGGCCCC
	181	CCCACTTTCC	TGGACACCCA	GGTAAAGTTC	AAGCTGGGCG	AGGAGTTCGA	CGAGTTCCCC
	241	AGCGACCGCC	GCAAGGGCGT	GAAGAGCGTC	GTGAACCTGG	TGGGGGAAAA	GCTGGTGTAT
	301	GTGCAAAAGT	GGGATGGGAA	GGAGACCACC	TACGTGCGCG	AGATCAAGGA	TGGCAAGCTG
	361	GTCGTGACCC	TCACCATGGG	CGACGTGGTG	GCCGTCCGCA	GCTACCGTCG	CGCCACCGAG
	421	TAA					
UnaGco (optimized)	1	ATGATTCTAG	AAAAATTTGT	TGGAACTTGG	AAGATCGCTG	ACTCTCATAA	TTTCGGCGAG
	61	TATCTCAAAG	CCATTGGAGC	TCCTAAGGAA	TTGTCAGATG	GCGGAGATGC	CACCACACCA
	121	ACTCTATACA	TCTCTCAAAA	AGATGGCGAC	AAGATGACCG	TGAAAATTGA	GAACGGACCT
	181	CCAACATTTC	TCGATACTCA	GGTAAAGTTC	AAATTGGGCG	AAGAGTTTGA	CGAATTCCCT
	241	TCAGATAGAC	GAAAGGGAGT	CAAATCTGTT	GTGAATCTAG	TAGGCGAGAA	GCTCGTCTAT
	301	GTTCAAAAAT	GGGACGGAAA	GGAAACCACA	TACGTGAGGG	AGATCAAAGA	TGGCAAGTTG
	361	GTAGTCACTC	TAACCATGGG	AGATGTTGTG	GCTGTAAGAT	CATATCGAAG	GGCCACAGAG
	421	TAA					
eGFP	1	ATGGTGAGCA	AGGGCGAGGA	GCTGTTCACC	GGGGTGGTGC	CCATCCTGGT	CGAGCTGGAC
	61	GGCGACGTAA	ACGGCCACAA	GTTCAGCGTG	TCCGGCGAGG	GCGAGGGCGA	TGCCACCTAC
	121	GGCAAGCTGA	CCCTGAAGTT	CATCTGCACC	ACCGGCAAGC	TGCCCGTGCC	CTGGCCCACC
	181	CTCGTGACCA	CCCTGACCTA	CGGCGTGCAG	TGCTTCAGCC	GCTACCCCGA	CCACATGAAG
	241	CAGCACGACT	TCTTCAAGTC	CGCCATGCCC	GAAGGCTACG	TCCAGGAGCG	CACCATCTTC
	301	TTCAAGGACG	ACGGCAACTA	CAAGACCCGC	GCCGAGGTGA	AGTTCGAGGG	CGACACCCTG
	361	GTGAACCGCA	TCGAGCTGAA	GGGCATCGAC	TTCAAGGAGG	ACGGCAACAT	CCTGGGGCAC
	421	AAGCTGGAGT	ACAACTACAA	CAGCCACAAC	GTCTATATCA	TGGCCGACAA	GCAGAAGAAC
	481	GGCATCAAGG	TGAACTTCAA	GATCCGCCAC	AACATCGAGG	ACGGCAGCGT	GCAGCTCGCC
	541	GACCACTACC	AGCAGAACAC	CCCCATCGGC	GACGGCCCCG	TGCTGCTGCC	CGACAACCAC
	601	TACCTGAGCA	CCCAGTCCGC	CCTGAGCAAA	GACCCCAACG	AGAAGCGCGA	TCACATGGTC
	661	CTGCTGGAGT	TCGTGACCGC	CGCCGGGATC	ACTCTCGGCA	TGGACGAGCT	GTACAAGTAG
EGFPco (optimized)	1	ATGGTTTCCA	AGGGGGAGGA	GCTATTTACA	GGTGTCGTCC	CTATTCTTGT	TGAATTGGAC
	61	GGAGATGTCA	ATGGACACAA	ATTTTCTGTC	TCCGGGGAGG	GAGAGGGAGA	CGCAACATAT
	121	GGCAAACTCA	CTCTCAAGTT	TATCTGCACC	ACTGGCAAGT	TACCTGTGCC	ATGGCCTACG
	181	CTTGTGACTA	CTCTAACATA	TGGTGTGCAA	TGTTTCTCTC	GCTATCCAGA	CCACATGAAA
	241	CAACACGATT	TTTTCAAATC	AGCTATGCCT	GAAGGGTATG	TTCAAGAGAG	GACTATCTTT
	301	TTCAAAGACG	ACGGAAATTA	TAAGACCAGA	GCAGAAGTCA	AATTCGAAGG	TGATACTCTG
	361	GTGAATCGAA	TCGAACTTAA	GGGTATTGAC	TTTAAGGAAG	ATGGTAATAT	TTTGGGCCAC
	421	AAATTAGAGT	ATAACTATAA	CTCTCATAAT	GTCTATATTA	TGGCAGACAA	ACAAAAAAAC
	481	GGGATAAAGG	TGAATTTTAA	AATTAGACAT	AACATCGAAG	ATGGGTCAGT	TCAACTCGCT
	541	GACCATTATC	AACAGAATAC	TCCTATCGGT	GATGGTCCTG	TTCTACTCCC	TGATAATCAC
	601	TATCTATCCA	CTCAATCAGC	TCTCTCAAAA	GACCCAAATG	AGAAAAGGGA	CCACATGGTG
	661	TTGCTAGAGT	TTGTTACTGC	GGCAGGAATT	ACACTAGGAA	TGGACGAGCT	ATATAAGTAG

Sequence optimization of eGFP (2120-T2A-eGFPco-2820) allowed for RC DNA formation, but with a shortened length. 2120-T2A-eGFPco-2820 formed a band, “f,” which electrophoresed slightly faster than the RC DNA of Pch9-G2016T (Figure 9A, lane 21). Notably, this band disappeared after *Eco*RI digestion and was suggested to be a shortened RC DNA from a spliced RNA. However, we reported a substantial intact pgRNA in samples of 2120-T2A-eGFPco-2820 (Figure 10A, lane 22), demonstrating potential defects in DNA synthesis. It is possible that the band, “g,” is the intact SS DNA, which is reverse transcribed from the intact pgRNA of 2120-T2A-eGFPco-2820 (Figure 9A, lanes 21 and 22).

## Discussion

Previous studies indicate that HBV recombinant virus expressing foreign genes can be constructed successfully. However, a systematic exploration of the influence of different strategies of engineering on HBV replication is lacking. In the present study, the whole genome of HBV was scanned for regions suitable for engineering. “Suitable here” means supporting the formation of RC DNA, the precursor of functional cccDNA. This criterion allowed for the identification of two regions, 2118–2814 and 99–1198. Region 2118–2814 covers the C-terminal of HBC and the N-terminal of TP domain of Pol, and its deletion efficiently supports RC DNA formation. Notably, this region partially overlaps the region (nt 2,124–2,712) previously utilized to recombine NanoLuc into an HBV of genotype C (accession number AB246345) (Nishitsuji et al., 2015). Region 2118–2814 can hardly extend further because the hM (2820–2868) region must be retained. Previously, researchers successfully inserted a 52-aa polypeptide into 1982–2312 (Wang et al., 2002, 2014; Deng et al., 2009). Even so, we think that extending the recombination region toward the N-terminal of HBC is undesirable. In the present analysis, deletion of 1919–2515 formed only weak RC and DL DNA, and the relatively lower level of core DNA was potentially associated with a low amount of intact pgRNA. Intriguingly, 1919–2515 deletion resulted in almost complete pgRNA splicing. A similar phenomenon was evident for D2899-197, which formed only weak SS DNA and showed a large part of pgRNA splicing. Elsewhere, a study revealed that 55% pgRNA of wild-type HBV suffered from splicing in HepG2 cells (Abraham et al., 2008). Taken together, significantly higher splicing of D1919-2515 and D2899-197 implies that the two regions are functional to protect authentic pgRNA from splicing. It is speculated that such protection is potentially associated with secondary or higher-order RNA structures that can hide the splicing sites.

Region 99-1198 covers the C-terminal of the spacer, RT, and RNaseH domains of Pol, overlapping preS2 and S ORF. In a previous study, BSD was inserted into the preS2 region, between *Xho*I (C^TCGAG, nt 125) and *Bsr*GI (T^GTACA, nt 766) (Liu et al., 2009). Moreover, Untergasser et al. inserted GFP between nt 1,446 and 2,347 (Untergasser and Protzer, 2004). This region contains h5E (nt 1511–1568), which has been reported to play a crucial role in RC DNA formation (Lewellyn and Loeb, 2007). The deletion of h5E (nt 1,511–1,568) was responsible for the poor RC DNA formation in this construction of HBV. Preserving h5E (nt 1,511–1,568), region 99–1198 is efficient for foreign genes recombination with a size < 1,000 bp.

Herein, in D3099-396, an additional band was detected between the RC and DL DNA. This band was likely pseudo-RC DNA, reverse transcribed from one of the spliced pgRNAs of D3099-396, because only spliced RC DNA could electrophorese faster than the unspliced RC DNA and slower than the unspliced DL DNA. Of note, all spliced pgRNAs of D3099-396 lacked hM (2820–2868), which was reported to be crucial for RC DNA formation (Lewellyn and Loeb, 2007).

Furthermore, we fused 10 foreign genes, via T2A peptides, into the ORF of HBC and HBS, at 2120 and 155, respectively. This in-frame arrangement allowed for the expression of foreign genes from two RNAs, preC RNA and pgRNA, or preS1 mRNA and S mRNA. T2A peptide fused at the N-terminal of the foreign genes reduced the potential impact of the fused HBV peptides on the function of foreign genes. The findings demonstrate that site 155 may be more tolerable to recombination than site 2120. Of the 10 genes, 9 were successfully inserted at site 155 without abolishing RC DNA formation, whereas 5 of the 10 genes inserted at site 2120 abrogated that. Besides, the deletion of HBV 2121-2819 did not abort RC DNA formation. Therefore, the failure of RC DNA formation of insertions of Neo, copGFP, mCherry, UnaG, and eGFP at 2120 was unlikely to be associated with deleting any *cis*-elements. Notably, the recombination of these genes still allowed for DL DNA formation. As such, the genes must interfere with the plus-strand primer translocation or the circulation step. Inserting the foreign genes close to hM (nt 2,820–2,868) implies that this arrangement could interfere with the base pairing between hM and h3E. These events are crucial for the translocation of plus-strand primer (Lewellyn and Loeb, 2007), contributing to the abortion of RC DNA formation.

The most intriguing finding of this work is that optimization of the sequence of recombinant genes may improve RC DNA formation. Usually, codon optimization is used to improve the expression of proteins as this approach accommodates the codon bias of the host organism. However, we do not think the alteration in protein (UnaG) expression ameliorated RC DNA formation in the present case. First, the HBC and Pol were provided by transcomplementation. Second, the expression of UnaG or UnaGco was not essential for RC DNA formation. Third, 2120-T2A-UnaG-2607 produced a higher level of pgRNA and SS DNA than 2120-T2A-UnaGco-2607. These findings relay evidence that UnaG recombination has no adverse effects on the steps preliminary to synthesizing minus-strand DNA. However, it is plausible that UnaG impeded RC DNA formation by limiting the step of plus-strand synthesis or circularization at the DNA sequence level, for example, through the formation of some secondary structures. In line with this, structure prediction of the minus-strand DNA showed that wild-type UnaG sequence does disrupt the original structure of minus-strand DNA, whereas UnaGco profoundly ameliorates this impact (Figure 11). An interesting implication of our findings is that the primary sequence of minus-strand DNA intrinsically serves as a selection pressure during evolution. That is, mutations adversely impacting the secondary structure of minus-strand DNA would have less fitness and be weeded out.

## Conclusion

In conclusion, the current study provides an informative basis and a valuable method for constructing and optimizing recombinant HBV. Efforts are being taken to obtain and characterize reporter HBV based on the recombinants constructed in the study.

## Data Availability Statement

The original contributions presented in the study are included in the article/supplementary material, further inquiries can be directed to the corresponding author/s.

## Author Contributions

C-YG contributed to the implementation of the research. JC, W-LZ, Y-WW, A-LH, and J-LH contributed to the design of the research, the analysis of the results, and the writing of the manuscript. All authors contributed to the article and approved the submitted version.

## Conflict of Interest

The authors declare that the research was conducted in the absence of any commercial or financial relationships that could be construed as a potential conflict of interest.

## Publisher’s Note

All claims expressed in this article are solely those of the authors and do not necessarily represent those of their affiliated organizations, or those of the publisher, the editors and the reviewers. Any product that may be evaluated in this article, or claim that may be made by its manufacturer, is not guaranteed or endorsed by the publisher.

## Footnotes

1
https://imagej.nih.gov/ij/docs/menus/analyze.html#gels
